# Dr. Charles Cook Ransom

**Published:** 1910-10

**Authors:** 


### OBITUARY

Dr. Charles Cook Ransom, of New York and Richfield Springs, died at a hospital in Utica, September 13, 1910, after a brief illness. He was 50 years old and a native of Richfield Springs. He was graduated from the Medical Department of the University of Buffalo in 1883.

